# Exclusive breastfeeding at 6 months after assisted and spontaneous conceiving: a prospective study in Northern Italy

**DOI:** 10.1038/s41598-023-33688-w

**Published:** 2023-04-20

**Authors:** Camilla Pisoni, Francesca Garofoli, Annalisa De Silvestri, Elisa Civardi, Stefano Ghirardello

**Affiliations:** 1 Neonatal Unit and Neonatal Intensive Care Unit, Fondazione IRCCS Policlinico San Matteo, Pavia, Italy; 2 Biometry & Clinical Epidemiology, Scientific Direction, Fondazione IRCCS Policlinico San Matteo, Pavia, Italy

**Keywords:** Nutrition, Paediatrics

## Abstract

Conceiving by assisted infertility treatments may influence breastfeeding duration. In one-year time, to evaluate the goal of 6 months breastfeeding, we recruited 55 consecutive mothers who conceived using assisted treatment compared to 45 mothers conceiving naturally, all giving birth to healthy, full-term, singleton infants, sharing the double-occupancy room. At birth, maternal/neonatal characteristics were obtained by medical records and interviews. Six months after, a telephonic interview was done about the exclusivity of breastfeeding, mood instability, and breastfeeding complications. All the women were supported by the same neonatal-pediatrician team, during the study period. The number of mothers who were exclusively breastfeeding at six months was not statistically different between the two groups, as well as, breastfeeding initiation, BMI, smoking habit, mood instability, co-morbidities. In the assisted group, the women were older, had fewer previous children, upper degree of education, higher rate of cesarean sections, their neonate’s birthweight was lower; they reported more breastfeeding complications, but the distribution was not different between groups. The control women had higher number of previously breastfed siblings. Our experience highlights that the mode of conception may not be the defining factor influencing the goal of 6 months lactation. The support of healthcare professional team has a crucial role in maintaining breastfeeding.

## Introduction

Breast milk has been classified as the gold standard for infant nutrition during early postnatal life. For these reasons, the World Health Organization (WHO), and most International Organizations, dealing with health and growth agreed that breastfeeding should start no later than one hour after birth and be kept as exclusive feed for at least 6 months^1–4^. Specifically, breastfeeding for the first six months of life decreases the risk of obesity, type 2 diabetes (T2D), and other non-communicable chronic diseases in the infant^1–5^. Based on the data assessed in 2005, the WHO set a target of 50% of infants exclusively breastfed worldwide until six months of age by 2025. However, in 2020, the global infant population who resulted solely breastfed until six months of life was 44%, making the WHO target challenging to achieve in 2025^5^. For most mothers, return to employment, lack of sustenance from health care providers, and physical challenges are the main reasons for quitting breastfeeding earlier than intended^6^.

During recent decades, infertility rates increased, especially in high-income countries where it is estimated that up to 15% of couples are infertile and have access to assisted reproductive treatment (ART)^7,8^. In Europe, 1 in 50 children results from ART treatments; in 2014 in Italy, 10.976 babies were born through ART, accounting for 2,2% of annual births^9^.

The impact of infertility treatments on the initiation and duration of breastfeeding is a critical issue to explore. Understanding the possible mechanism of action for shorter duration of breastfeeding following fertility treatment is not yet established, but recognizing the major cause could allow us to find strategies to improve breastfeeding itself. Literature highlight controversial results about breastfeeding duration after assisted pregnancy, in particular if this association exists or not, and if depends by different types or length of fertility intervention. Some authors demonstrated that women who conceived through ART stopped breastfeeding earlier or breastfed for a shorter period than those who conceived spontaneously; other authors showed no differences in the length of breastfeeding between spontaneous and fertility treated conceiving^8–17^.

In addition, women who undergo fertility treatments often experience different levels of stress and anxiety, and it is well known that poor maternal mental and emotional health may have a negative impact on breastfeeding^18^. Conceiving fertility treatment thus had contradictory effects on maternal milk production, but the number of available studies is scarce.

Our prospective study aimed to evaluated the exclusivity of breastfeeding at six months of neonate's life and the influence of the mode of conception, simultaneously investigating pairs of roommate mothers, supported by the same neonatal-pediatrician team, in 1 year time.

## Materials and methods

### Patients

This prospective study evaluates consecutive mothers who conceived using ART, giving birth to healthy, full-term (gestational age (GA) > 37 weeks, with body-weight between 10° and 90° percentile) singleton infants staying in the Neonatal Unit at IRCCS Fondazione Policlinico S. Matteo (Italy) from September 2018 to September 2019. ART treatments included intra cytoplasmic sperm injection (ICSI), in vitro fertilization and embryo transfer (IVF-ET), intrauterine insemination (IUI), and ovarian stimulation. Controls were the mothers who naturally conceived healthy, full-term singleton newborns and simultaneously shared the same double-occupancy room in the post delivery period. The local Ethical Committee has approved the study (Prot 20170001956 June 9, 2017) and all the mothers gave written informed consent to participate.

### Procedures

During the hospitalization in Neonatal Unit after delivery, a trained staff, consisting of a researcher, a medical resident in pediatrics and a nurse, proposed women to participate in the study and asked to sign informed consent. Trained staff collected maternal, and neonatal socio-demographic characteristics during the hospital stay using medical records and direct interviews with study-specific fixed-choice questions. In addition, six months after delivery, a telephonic interview was done about the exclusivity of breastfeeding, mood instability and breastfeeding complications. The same experienced neonatal and pediatrician team was involved throughout the study. Moreover, all neonatal team involved in postpartum promoted practices supporting breastfeeding, and sustained women even after discharge.

### Outcome measures

Data recorded at birth were as follows:

Characteristics of patients: age at delivery, body mass index (BMI), socio-professional category, as Married/partner, < High School/ ≥ High School, Employed/Unemployed/housewife, smoking during pregnancy, coffee habit. Incidence of co-morbidities during pregnancy (gestational diabetes, hypertension, thyroid pathologies, HELLP syndrome, eclampsia, pre-eclampsia), mood instability before and after the delivery. Previous conceiving, ART/natural, breastfeeding in case of prior children. Delivery mode, breastfeeding initiation.

Singleton pregnancy and healthy full-term offspring were inclusion criteria.

A telephone interview concerning breastfeeding exclusivity was performed six months after the delivery. Mothers were asked about mood instability, the question was: “Do you have frequent ups and downs in mood, which happen for no reason and give you problems in everyday life?”.

Then mothers, were also asked about complication during breastfeeding; these latter were grouped into three categories: maternal conditions (tiredness, breast pain); lactation problems (poor suckling, scarce milk); working/social needs.

### Statistics

Categorical variables were described as counts and percentages and compared between groups with the Chi-square test. Continuous variables were expressed as mean and standard deviation (SD) or median and Interquartile range (IQR 25%-75%); quantitative variables as mean and SD if normally distributed (Shapiro–Wilk test), with median and interquartile range otherwise. Comparisons between groups were made with T-test for independent data or the Mann–Whitney test.

Univariate logistic regression models were fitted using six months of full maternal milk lactation as a dependent variable and maternal and neonatal characteristics, reported in Tables 1 and 2 as independent factors. Univariable logistic regression analysis was used to test the association of one explanatory variable at a time with the outcome of interest. Those with a *p*-value < 0.20 were entered into a multivariate model to obtain adjusted effects. Results are expressed as odds ratio (OR) and are reported with 95% Confidence interval (95% CI). A *p*-value < 0.05, two-sided was considered statistically significant. Data analysis was performed with the STATA statistical package (StataCorp. 2019. Stata Statistical Software: Release 17. College Station, TX: StataCorp LLC).

**Table 1:** Maternal characteristics.

	ART n = 55	Controls n = 45	*P* value
Age, Years, mean (SD)	36,09 (4,27)	32,49 ( 5,19)	**0.001**
BMI before pregnancy, mean (SD)	22,54 (4,7)	22,74 (3,72)	0.82
Married/partner Number (%)	53 (96)	40 (89)	0.15
Number (%) < High School	27 (49)	31 (69)	**0.046**
≥ High School	28 (51)	14 (31)	
Number (%) Employed,	47 (85.5)	34 (75.5)	0.21
Unemployed/housewife	8 (14.5)	11 (24.5)	
Smoking during pregnancy Number (%)	0	0	–
More than 2 coffees/day Number (%)	23 (42)	23 (51)	0.41
Hypertension, Number (%)	6 (11)	3 (6.5)	0.46
Diabetes, Number (%)	7 (13)	4 (9)	0.46
Thyroid abnormalities, Number (%)	18 (33)	9 (20)	**0.017**
Pregnancy, mood instability, Number (%)	10 (18)	7 (15.5)	0.73
Siblings (YES) Number, (%)	14 (25.5)	28 (62)	**0.000**
Previous conceiving, Number, (%) ART	4 (28.5)	0	**0.000**
Natural	10 (71.5)	28 (100)	
Breastfeeding of previous children Number, (%)	10 (71.5)	24 (86)	0.078
Delivery, Number (%) Vaginal	29 (53)	38 (84)	**0.041**
Caesarian	26 (47)	7 (16)	
Breastfeeding before 4 h	36 (65.5)	37 (82)	0.06
Breastfeeding after 4 h	19 (34.5)	8 (18)	
Number (%)			
Post-natal, mood instability, at 6 months Number (%)	22 (40)	11 (24.5)	0.10
Breastfeeding difficulties, at 6 months (total) Number (%)	41 (74.5)	21 (53.3)	**0.004**
Maternal conditions (tiredness/breast pain)	22 (58.6)	15 (71.4)	0.25
Lactation problems (poor suckling/ scarce milk)	10 (24.3)	2 (9.5)	
Working/social needs	1 (0.5)	1 (4.7)	
More than 1 problem of the above	8 (4.4)	3 (5.5)	
Exclusive breastfeeding at 6 months, Number (%)	9 (16)	14 (31)	0.081

Significant values are presented in bold.

**Table 2:** Neonatal characteristics.

	ART = 55	Controls n = 45	*P* value
Gestational age, weeks Mean (SD)	39,07 (1,12)	39,33 (1,22)	0.27
Birthweight, (g) Mean (SD)	3108,27 (401,1)	3328,56 (370,92)	**0.005**
Number (%) Male	31(56)	21 (46.5)	0.33
Female	24(44)	24 (53.5)	

Significant values are presented in bold.

### Ethics approval and consent to participate

The study was conducted in accordance with the Helsinki declaration for investigations in human subjects. The Ethical Committee of IRCCS Policlinico S. Matteo, Pavia, Italy has approved the protocol (p- 20170007595; prot 200170001956). All the patients signed the consent form for the anonymous use of clinical data for scientific purposes, approved by the Ethical Committee.

## Results

During the one-year study period, we enrolled 55 mothers in the ART group and 55 controls; 10 control mothers refused or were not available to participate in the follow-up survey, for personal reasons. Maternal characteristics are reported in Table 1: Maternal characteristics.

The number of mothers who were still exclusively breastfeeding at six months of age was not statistically different between groups (*p* = 0.081; 16% ART vs. 31% controls) as well as the early time of initiation of breastfeeding (*p* = 0.060). The birthweigh of the neonates born to ART mothers was statically lower than newborns to spontaneous conceiving women (*p* = 0.005). Table 2 describes neonatal characteristics.

Compared to the control group, mothers who conceived with fertility treatment were older, had fewer previous pregnancies, had an upper degree of education, and had a higher rate of cesarean section (*p* < 0.001 for all comparisons). In addition, spontaneously conceiving women had a significantly higher number of previously breastfed siblings (*p* < 0.001, both). The two groups were statistically similar in BMI, smoking habit, the incidence of co-morbidities (gestational diabetes, hypertension, thyroid pathologies, HELLP syndrome, eclampsia, pre-eclampsia), and mood instability before and after the delivery (Table 1). However, mothers of the ART group suffered from complications in maintaining breastfeeding, the number of problems they reported was statically higher (*p* = 0.004; 74.5% ART vs. 53.3% controls). Nevertheless, the distribution frequency was not significant (*p* = 0.25) compared to the control group (Table 1).

In the ART group, the cesarean section (*p* = 0.041) and thyroid dysfunction (*p* = 0.017) were negatively related to the target of six months of exclusive maternal milk lactation compared to the spontaneous conceiving group. Nevertheless, these associations were no longer statistical significant performing the multivariate analysis (Odds Ratio caesarian section: 0.278; IQR 25–75%: 0.072–1.068; *P* > 0.062. Odds Ratio thyroid dysfunction: 0.540; IQR 25–75%: 0.159–1.831; *P* > 0.323).

In Table 3 we reported a multivariate model to test the association of one explanatory variable at a time with the outcome of interest, those with a *p*-value < 0.20, considering exclusive breastfeeding (EBF) at 6 months, as dependent variable.

**Table 3:** Multivariate logistic regression model fitted using six months of exclusive breastfeeding (EBF) as dependent variable and maternal characteristics with *p* < 0.20 at univariate analysis as independent factors.

EBF	OR	95% CI	*p*
Mode of conceiving	0.63	0.23–1.73	0.371
Mode of delivery	0.28	0.07–1.7	0.062
Tyrhoid abnormalities	0.54	0.16–1.83	0.323

OR: odds ratio; 95% CI: 95% confidence interval.

## Discussion

The growing number of couples conceiving through fertility treatments raised the necessity to explore whether different needs and outcomes belong to this population. Our study showed that the mode of conception did not statistically influence the capacity to comply with the first important goal defined by WHO, and other Organizations, precisely consisting of 6 months of exclusive maternal lactation, even if there is an important difference within the sample numbers^2–4^. We found that, in the ART group, cesarean section and thyroid dysfunction were negatively associated with 6-month exclusively breastfeeding. Indeed, it is known that women who give birth by caesarean section are less likely to initiate lactation timely and report more difficulties establishing and continuing breastfeeding^15,19^. Similarly, a clear negative association between hypothyroidism and breastfeeding has been reported due to the suppression of milk in mothers with this dysfunction^20^; nevertheless, in the multivariate analysis, these associations were no longer significant after adjusting for all the covariates.

Two previous Italian case–control studies^10,16^ that examined the association between the mode of conception and breastfeeding outcomes obtained similar results. Cromi et al.^16^ investigated the initiation, duration and exclusivity of breastfeeding among women who had conceived with the help of ART, compared with a matched control group who conceived spontaneously. Cases were as likely as controls to initiate breastfeeding, but by six weeks postpartum, a higher proportion of mothers who conceived through ART stopped breastfeeding. Then, the percentage of mothers who exclusively breastfed their child for six months became similar between the two groups. Pileri et al.^10^ described similar results for ART and physiologically conceiving mothers. The length and exclusivity of breastfeeding were mainly associated with the multidisciplinary maternal breastfeeding approach, such as information, promotion and support, but not with the mode of conception. On the other hand, further studies from different countries^11–13,15,17,21^ concluded that fertility treatment was associated with shorter breastfeeding duration, possibly attributable of multiples and infants born preterm.

An Iranian^13^ study demonstrated that ART conception was associated with worse breastfeeding outcomes determined by a lower rate of exclusive breastfeeding, a higher rate of breastfeeding dysfunction, and bad feeling during breastfeeding. A Chinese^15^ study showed a breastfeeding period shorter than six months for mothers who conceive through ART compared to mothers who conceive spontaneously; nevertheless, the initiation of lactation was comparable. In the USA, the study of Barrera et al.^11^ evidenced that mothers who conceive using ART breastfed for shorter durations, considering demographic and health covariates. Still, this difference was no longer significant after adjusting for plurality and preterm birth. These differences may depend on studies' methodological limitations, which includes inadequate recruitment, lack of appropriate comparison groups and inappropriate adjustment for potential causal intermediates such as preterm birth or multiple births^14^. Indeed, Weng et al. in a study conducted in a sample of 3465 women, highlighted that the effects of mode of conception on breastfeeding outcomes became insignificant in cases of exclusive singleton birth comparison^21^.

However, as reported by WHO, socio-economic and cultural influences have a profound impact on breastfeeding, which described an inverse relationship between breastfeeding at six months and the log gross domestic product per person in sub-Saharan Africa, south Asia, and Latin America, compared to most high-income countries^22,23^. This contrasting evidence may support the conclusion that the length of exclusive human milk nutrition depends not on the different modes of conception but mostly on socioeconomic and cultural differences, with higher education levels and financial possibilities associated with more prolonged breastfeeding. Moreover, concurring with other studies, we may think that women's decisions and experiences about feeding infants are influenced by the intention to breastfeed rather than the way of conceiving^9,16^. Exclusive breastfeeding at 6 months could also compensate for the potential emotional damage caused by not being able to conceive naturally; as a result, some might attempt to breastfeed their infant for as long as possible^24^. Furthermore, women who undergo fertility treatments are generally described as task-driven and goal-oriented because fertility treatments require them to be so. These personality traits and the strong desire to breastfeed, considered a maternal skill, may have affected ART mothers' drive to succeed at breastfeeding^25^. In addition, confirming this hypothesis, our ART sample successfully copes with more difficulties during breastfeeding; they suffered from more complications in maintaining breastfeeding, without affecting its duration. Moreover, the latest Italian survey, between December 2018 and April 2019, remarks that less than one quarter (23.6%) of children aged 4–5 months were exclusively breastfed, presenting differences between geographical areas^26^. Thus, our results about the prevalence of breastfeeding (16% ART vs. 31% controls) fit the above-cited national data. As with other authors, we reported that neonates from ART women had a significantly lower bodyweight than controls newborns at birth^27^.

Our study presents some critical issues. First, it could be important to investigate in depth women's mental health. We chose to use the trans-diagnostic concept of mood instability, which is a prominent feature of women in their reproductive years, particularly during pregnancy and postpartum^28^. It would also have been appropriate to investigate anxiety and depression with specific questionnaires, to better describe the women's mental health and highlight any links with breastfeeding.

Finally, we are aware of limited number of mother/baby couples, that is the principal limitation of the present study, nevertheless, we think that this can be the a solid picture of our local reality, in Northern Italy considering one year period. The effort we made to have comparable groups with homogeneous clinical characteristics (mothers sharing the double-occupancy room simultaneously, who gave birth to healthy, full-term, singleton infant) and the pragmatic enrollment, coupled with the support given by the same formed team, makes the obtained results reliable. Moreover, these outcomes comply with the previous cited Italian studies^10,16^. Nevertheless, we believe that our study can be further confirmed in a larger investigation.

Our survey highlighted that the mode of conception may not be the defining factor influencing the capacity to comply with the critical goal of 6 months of exclusive maternal lactation. We underline that the support by healthcare professional team has a crucial role in maintaining breastfeeding, regardless of the way of conception.

## Data Availability

The datasets used and/or analyzed during the current study are available from the corresponding author on reasonable request.
